# Detection of carcinoembryonic antigen messenger RNA in blood using quantitative real-time reverse transcriptase-polymerase chain reaction to predict recurrence of gastric adenocarcinoma

**DOI:** 10.1186/1479-5876-8-107

**Published:** 2010-10-31

**Authors:** Miao-zhen Qiu, Zhuang-hua Li, Zhi-wei Zhou, Yu-hong Li, Zhi-qiang Wang, Feng-hua Wang, Peng Huang, Fahad Aziz, Dao-yuan Wang, Rui-hua Xu

**Affiliations:** 1 State Key Laboratory of Oncology in South China, Guangzhou 510060, China; 2 Department of Medical Oncology, Sun Yat-Sen University Cancer Center, Guangzhou 510060, China; 3 Department of GI Surgery, Sun Yat-Sen University Cancer Center, Guangzhou 510060, China; 4 Department of Molecular Pathology, The University of Texas, MD Anderson Cancer Center. USA; 5 Jersey City Medical Center, Mount Sinai School of Medicine, NY. USA; 6 AmMed Cancer Center, Shanghai Ruijin Hospital, Medical School of Shanghai Jiaotong University, Shanghai 200035, China

## Abstract

**Background:**

The existence of circulating tumor cells (CTCs) in peripheral blood as an indicator of tumor recurrence has not been clearly established, particularly for gastric cancer patients. We conducted a retrospective analysis of the relationship between CTCs in peripheral blood at initial diagnosis and clinicopathologic findings in patients with gastric carcinoma.

**Methods:**

Blood samples were obtained from 123 gastric carcinoma patients at initial diagnosis. mRNA was extracted and amplified for carcinoembryonic antigen (CEA) mRNA detection using real-time RT-PCR. Periodic 3-month follow-up examinations included serum CEA measurements and imaging.

**Results:**

The minimum threshold for corrected CEA mRNA score [(CEA mRNA/GAPDH mRNA) × 10^6^] was set at 100. Forty-five of 123 patients (36.6%) were positive for CEA mRNA expression. CEA mRNA expression significantly correlated with T stage and postoperative recurrence status (*P *= 0.001). Recurrent disease was found in 44 of 123 cases (35.8%), and 25 of these (56.8%) were positive for CEA mRNA. Of these patients, CEA mRNA was more sensitive than serum CEA in indicating recurrence. Three-year disease-free survival of patients positive for CEA mRNA was significantly poorer than of patients negative for CEA mRNA (*P *< 0.001). Only histological grade and CEA mRNA positivity were independent factors for disease-free survival using multivariate analysis.

**Conclusions:**

CEA mRNA copy number in peripheral blood at initial diagnosis was significantly associated with disease recurrence in gastric adenocarcinoma patients. Real-time RT-PCR detection of CEA mRNA levels at initial diagnosis appears to be a promising predictor for disease recurrence in gastric adenocarcinoma patients.

## Background

Gastric cancer remained the leading cause of cancer mortality worldwide throughout the 20th century. The only proven curative treatment is surgical resection of all gross and microscopic lesions. However, despite undergoing curative gastrectomy, including extended lymph node dissection and adjuvant chemotherapy, cancer recurs in both regional as well as distant sites in majority of the patients [1]. Diagnosis of recurrence with common follow-up protocols usually is made at a late stage, which, to an extent, precludes the possibility of effective treatment [2]. Surveillance of circulating tumor cells (CTCs) seems to offer greater possibility for earlier diagnosis of recurrent disease.

The concept of investigating the metastatic process in peripheral blood originated in the 19th century when T.R. Ashworth first described the phenomenon of CTCs, and S. Paget hypothesized a non-random pattern of cancer metastasization (the 'seed and soil' theory) [3,4]. Subsequently, the malignant nature of CTCs was confirmed by demonstrating that they possess tumor-specific chromosomal aberrations [5,6] and that they grow ex vivo as cell lines with a malignant phenotype [7]. Several approaches to detect CTCs have been described and can be classified into PCR-based methods and cytometric methods [8].

With the advent of quantitative real-time PCR techniques [9], precise quantification of a target sequence has become possible. Quantitative PCR provides investigators not only with technical advantages, but also with applicative advantages, such as the definition of cutoff values indicating mRNA expression levels of clinical relevance in cancer patients compared with healthy subjects. Real-time PCR also affords the possibilities of correlating target-sequence load with clinical outcome [10] or response to therapy [11].

CEA, originally described as a tumor-associated colon cancer antigen, was cloned in 1987 and is now recognized as a member of the immunoglobulin protein superfamily [12]. Many studies have reported detection of gastric cells in blood [13], bone marrow [14], and peritoneal washing [15] of gastric cancer patients by using real-time PCR for CEA mRNA.

The goal of this study was to evaluate the effectiveness of the CEA mRNA real-time PCR technique for the early detection of tumor recurrence. To meet this goal, the relationship between clinical recurrence and blood levels of CEA mRNA preoperatively was examined in gastric adenocarcinoma patients.

## Methods

### Patients

Written informed consent was obtained from every patient on the use of blood samples for research in accordance with the institutional guidelines of our hospital. Between February 2002 and December 2006, a total of 123 consecutive patients with gastric adenocarcinoma at Cancer Center of Sun Yat-sen University were enrolled into this study. All patients received radical resection and D2 lymphadenectomy. At lease 15 lymph nodes were available for the detection. No peritoneal dissemination was found. Clear records of serum CEA change and imaging evaluation before the operation and every three months after the operation were required. Patients who had positive lymph node were recommended to receive adjuvant chemotherapy but finally only eighty-three patients underwent adjuvant chemotherapy. The regimens included CAPOX (Capecitabine + Oxaliplatin, 16 cases, with a median cycle of 4), folfox6 (56 cases, with a median cycle of 6), taxol + cisplatin (4 cases, with a median cycle of 4), taxol + 5FU/CF (Fluorouracil/Leucovorin, 7 cases, with a median cycle of 6). Recurrent disease, including local relapse and distant metastases, was detected by computed tomography examination. New lesions detected by imaging examination in follow-up appointments were regarded as recurrence. Biopsy was not done routinely to determine histological recurrence. All imaging was evaluated by at least two independent observers, including radiologists. The median follow-up period was 37.0 months (range, 3.0-73.6 months).

### Blood samples

Blood samples were collected at initial diagnosis one or two days before surgery. The first 3 mL of blood was discarded to prevent epidermal contamination, and then a 5-mL blood sample was obtained from the peripheral vein. Peripheral blood samples obtained from 30 non-cancer patient volunteers were used as negative controls.

All patients provided written informed consent; we obtained separate consent for use of blood sample. Study approval was obtained from independent ethics committees at Cancer Center of Sun Yat-Sen University. The study was undertaken in accordance with the ethical standards of the World Medical Association Declaration of Helsinki.

### Pre-processing of blood samples

Blood samples were collected in EDTA-containing tubes. Sample processing was performed within 2 hours after blood withdrawal. Blood was transferred into a 30-mL falcon tube and centrifuged at 1,800 rpm at room temperature for 20 minutes. Serum was removed, and cells were resuspended in 5 mL saline and 0.3 mL RNA later solution. After mixing well, the blood cell mixture was kept overnight at 4°C and stored at -80°C until used.

### RNA extraction and cDNA synthesis

Total RNA of peripheral blood samples was extracted using RNAprep Cell Kit (Tiangen, Beijing, China) following the protocol provided by the manufacturer. RNA integrity was checked by electrophoresis and quantified by absorption at 260 and 280 nm using a UV-visible spectrophotometer (Beckman Coulter Du^® ^800, Fullerton, CA). For reverse transcription, 1 μg of total RNA, 1 μL Oligo(dT)15 and 1 μL dNTP were diluted in 10 μL RNase-free water, incubated 10 minutes at 37°C and 1 μL of 25 mmol/L EDTA was added. An 11 μL aliquot of reaction mixture was incubated for 10 minutes at 65°C and quickly chilled on ice for 2 minutes. cDNA was stored at -80°C until used. cDNA synthesis was performed using the TIANScript M-MLV method (Tiangen Biotech, Beijing, China).

### Cell lines

To prepare for CEA-specific RT-PCR, two cell lines, SW-480 (colon cancer cell line) and SC-7901 (gastric cancer cell line) were used. Lymphocytes were collected from healthy volunteers without epithelial malignancy. After lymphocytes were isolated from peripheral blood by gradient centrifugation, the mononuclear cell layer was collected. Cell lines were serially diluted (10-fold) in 2 × 10^7 ^to 5 × 10^7 ^lymphocytes to give carcinoma cell: lymphocyte ratios ranging from 1:10 to 1:10^7^.

### CEA mRNA Analysis by Real-Time Quantitative PCR

Quantitative PCR was performed using the Sequence Detector System, ABI PRISM 7500 (Applied Biosystems 7500 Fast Real-Time PCR System). PCR primers and the TaqMan probes were designed using the Primer Express 1.0 software program. In this assay, the housekeeping gene glyceraldehyde 3-phosphate dehydrogenase (GAPDH) was used as an internal control to normalize variations in integrity and total amount of RNA extracted. The real-time PCR assays for GAPDH and CEA were done in separate tubes. CEA mRNA values were adjusted against GAPDH mRNA values, and the relative CEA mRNA scores were presented as (CEA mRNA/GAPDH mRNA) × 10^6 ^for each sample.

5 μL of the sample cDNA was used for real-time PCR in a 20 μL reaction mixture consisting of 10 pmol appropriate primers (Invitrogen Cooperation, Japan) and 5 pmol TaqMan probe (Invitrogen Cooperation, Japan). The reporter dye (6-carboxy-fluorescein: FAM) was covalently attached to the 5' end of the probe, and the quencher dye (6-carboxy-tetramethyl-rhodamine: TAMRA) was attached to the 3' end of the probe. The temperature profile used for amplification was as follows: denaturation for 1 cycle at 95°C for 10 minutes, followed by 40 cycles at 95°C for 10 seconds, 60°C for 15 seconds, and 72°C for 5 seconds. Quantification was done by the ABI Prism 7500 Sequence detector system. Each set of samples and serially-diluted external controls were amplified in duplicate. The average value of the duplicates was used as the quantitative value.

A CEA-specific oligonucleotide primer was designed based on the report by Gerhard *et al. *[16]. The sequences were: 5'-TGTCGGCATCATGATTGG-3' (sense) and 5'-GCAAATGCTTTAAGGAAGAAGC-3' (antisense). Fluorescent and LC-Red probe sequences used for CEA identification were: 5'-CCTGAAATGAAGAAACTACACCAGGGC-fluorescein and 5'-LC-Red 640-GCTATATCAGAGCAACCCCAACCAGC-phosphate.

Real-time PCR monitoring was achieved by measuring the fluorescence signal at the end of annealing phase of each cycle. The primer sequences used for GAPDH amplification were: 5'-TGAACGGGAAGCTCACTGG-3'(sense) and 5'-TCCACCACCCTGTTGCTGTA-3' (antisense). The probe sequences used for GAPDH identification were: 5'-TCAACAGCGACACCCACTCCT-fluorescein and 5'-LC-Red 640-CACCTTTGACGCTGGGGCT-phosphate.

### Determination of CEA in serum samples

Pre-operative serum samples were also used for assaying tumor marker CEA using a commercially available enzyme immunoassay kit (Cobas Core EIA, Roche, Basel, Switzerland). Pathological cutoff level was established at 5 ng/mL for serum CEA.

### Statistical analyses

The Kaplan-Meier statistical method was used for analyzing clinical features and recurrence; differences were estimated with the log-rank test. Prognostic factors were examined by univariate and multivariate analyses (log-rank test for univariate analysis and Cox proportional hazards regression model, backward stepwise (conditional LR) for multivariate analysis). The chi-squared and Fisher exact tests were used for statistical analysis. All statistical analyses were done with SPSS16.0. All P values were 2-tailed, and the level of significance was set at 0.05.

## Results

### Clinical features

The 123 patients enrolled in the study aged 28 to 84 years (mean, 57.11 years; median, 59 years), and the ratio of males to females was 82:41 (Table 1). Staging was performed according to the Tumor-Node-Metastasis (TNM) classification of the American Joint Committee on Cancer (AJCC, revised 1997). Twenty-four tumors were located in the cardia, 3 in the gastric fundus, 44 in the gastric corpus, 45 in the gastric antrum, 5 involved the whole stomach, and 2 belonged to the remnant gastric carcinoma (Table 1).

**Table 1:** Clinicopathologic features and CEA* mRNA expression detected by real-time RT-PCR

Characteristics	Total number (N = 123)	CEA mRNA		P value
			
		Positive (n = 45)	Negative (n = 78)	
Age				
≤40	13	3	10	
41-50	20	9	11	
51-60	39	9	30	
61-70	42	18	24	
>70	9	6	3	0.063
Sex				
Female	41	12	29	
Male	82	33	49	0.234
Tumor				
T1	10	6	4	
T2	16	3	13	
T3	73	26	47	
T4	24	10	14	0.001
Lymph node				
N0	31	11	20	
N1	43	15	28	
N2	29	8	21	
N3	20	11	9	0.261
Pathologic TNM# stage				
I	16	7	9	
II	21	6	15	
III	51	17	34	
IV	35	15	20	0.623
Histology subtype				
Well differentiated adenocarcinoma	3	1	2	
Moderately differentiated adenocarcinoma	27	7	20	
Poorly differentiated adenocarcinoma	93	37	56	0.418
Serum CEA condition				
Positive	30	11	19	
Negative	93	34	59	0.992
Recurrence				
Yes	44	25	19	
No	79	20	59	0.001
Modes of recurrence				
Local recurrence	8	4	4	
Abdominal cavity	9	6	3	
Liver	8	5	3	
pelvic	6	3	3	
Multiple sites	10	5	5	
others	3	2	1	0.959

* Carcinoembryonic antigen

# TNM denotes tumor-node-metastasis

### Detection sensitivity of CEA mRNA by real-time RT-PCR

CEA mRNA was detected in SW-480 and SC-7901 cell lines. The lower limit of detection was a concentration of 10 tumor cells per 10^7 ^lymphocytes. Conventional nested RT-PCR was employed to confirm the sensitivity of the RT-PCR product.

### CEA mRNA expression in blood

**Figure 1:**
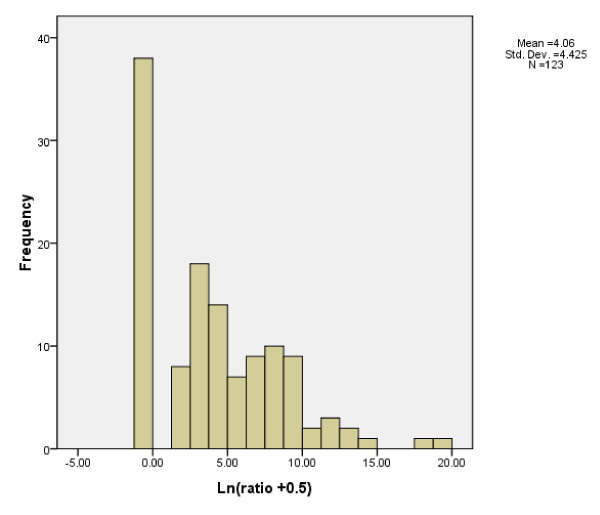
**The distribution of CEA expression level**. The ratio means (CEA mRNA/GAPDH mRNA) × 10^6^. Considering the ratio of some patients were zero, we added 0.5 to the ratio.

CEA mRNA expression was detected in 9 of 30 (30.0%) non-cancer patients, and the mean corrected CEA mRNA score was 7.5 (range, 0-92.5). The maximum value of corrected CEA mRNA score in patients without malignancy was 92.5, so a cutoff value of 100 was used in the present study. Using this cutoff value, 45 patients (36.6%) were diagnosed as CEA mRNA-positive. The mean corrected CEA mRNA score [(CEA mRNA/GAPDH mRNA) × 10^6^] of the 123 patients was 37,510.0 (range, 0-3,695,652.1) copies Figure 1 showed the distribution of (CEA mRNA/GAPDH mRNA) × 10^6 ^in this group of patients.

### Relationship between CEA mRNA expression and clinicopathologic features

CEA mRNA expression did not correlate with age, gender, N stage, TNM stage, histological subtype and serum CEA condition (Table 1). However, patients with postoperative recurrence had significantly higher percentage of CEA mRNA positive than those without tumor recurrence (*P *= 0.001) (Table 1). Besides, tumor depth also positively correlated with CEA mRNA expression (*P *= 0.001).

### Relationship between recurrence and CEA mRNA expression as well as serum CEA

The mode of recurrence includes 8 local recurrence, 9 abdominal dissemination except liver, 8 liver metastasis, 6 pelvic metastasis, 3 other sites metastasis and 10 multiple sites metastasis. There is no significant difference between the CEA mRNA expression and the mode of recurrence (Table 1).

Recurrent disease was found in 44 of 123 cases (35.8%). Twenty-five of these patients (56.8%) were CEA mRNA-positive. By contrast, only 14 patients with recurrent disease (31.8%) were positive for preoperative serum CEA. The specificities of CEA mRNA and serum CEA to indicate recurrence were 74.7% and 79.9%, respectively. (Table 2).

**Table 2:** Comparison between CEA* mRNA and serum CEA in predicting recurrence

		CEA mRNA		Serum CEA	
		
		Positive	Negative	Positive	Negative
Recurrence	Yes	25	19	14	30
	No	20	59	16	63
*P*		0.001		0.152	
*X*2		12.088		2.050	
Sensitivity (%)		56.8		31.8	
Specificity (%)		74.7		79.7	

* Carcinoembryonic antigen

### Univariate and multivariate analyses of 3-year disease-free survival

**Figure 2:**
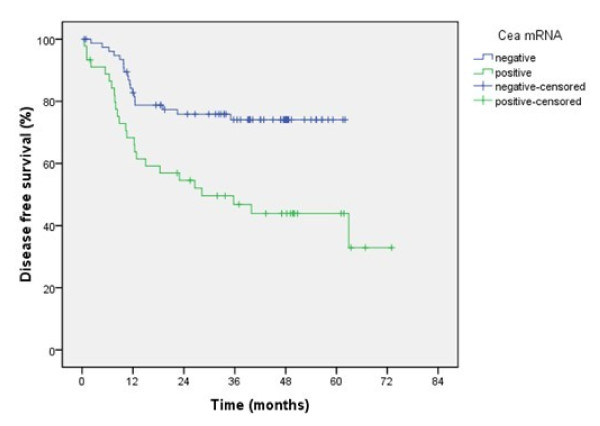
**Disease-free survival of patients according to CEA mRNA expression**. Three-year disease-free survival of CEA mRNA-positive patients was significantly lower than that of CEA mRNA-negative patients (43.9% versus 74.1%, respectively; *P *= 0.001).

The 5-year overall survival was 58.9% and 3-year disease free survival was 63.9%. Both univariate and multivariate analyses were used to evaluate factors relating to disease-free survival. According to univariate analysis, age, tumor depth, nodal metastasis, histological grade, TNM stage, CEA mRNA positivity and serum CEA positivity were significantly related to disease-free survival (*P *= 0.031, <0.001, 0.001, 0.022, <0.001, 0.001, 0.045 respectively) (Table 3). Multivariate regression analysis showed that only histological grade and CEA mRNA positivity were independent factors for disease-free survival (*P *= 0.047 and 0.020, respectively, Table 4). Three-year disease-free survival rates for CEA mRNA-positive patients were significantly lower than for CEA mRNA negative patients (43.9% versus 74.1%, respectively, *P *= 0.001, Figure 2).

**Table 3:** Univariate analysis of disease-free survival in Gastric carcinoma

Parameter	**Disease-free survival, P value **^**a**^
Age	
<59 vs. ≥59	0.031
Gender	
Male vs. female	0.433
CEA mRNA	
(+) vs. (-)	0.001
Serum CEA	
(+) vs. (-)	0.045
Histological grade	
Well/moderately vs. poorly	0.022
pT	
pTis/pT1 vs. pT2/pT3/pT4	<0.001
pN	
(+) vs. (-)	0.001
Stage	
1/2 vs. 3/4	<0.001
Adjuvant chemotherapy agents	
CAPOX vs. mFOLFOX6 vs. Taxol+DDP vs. Taxol+5Fu/CF	0.850

^a ^Log-rank test

**Table 4:** Multivariate analysis of disease-free survival in gastric carcinoma

Factors	Characteristics		Hazard ratio	95%CI	P value
				
	Unfavorable	Favorable			
Age	≥59	<59	1.018	0.986-1.050	0.273
Histological grade	Poorly	Well/moderately	0.412	0.171-0.990	0.047
pT	2/3/4	Tis/1	1.673	0.100-8.690	0.947
pN	(+)	(-)	2.030	0.264-15.611	0.497
Stage	3/4	½	1.437	0.124-16.613	0.771
CEA mRNA	(+)	(-)	2.243	1.138-4.424	0.020
Serum CEA	(+)	(-)	1.130	0.582-2.194	0.717

CI, confidence interval; pT, depth of tumor invasion

## Discussion

The semi-quantitative nature of traditional PCR technology has made it difficult to differentiate baseline gene expression levels in normal tissues from increased gene expression levels in cancer, thereby increasing the concern for false-positive results [17]. In our study, real-time PCR of CEA mRNA was used to investigate the possibility of peripheral blood as a source for CTC detection and prediction of cancer recurrence in gastric carcinoma patients. Real-time quantitative CEA mRNA analysis in cancer patients is often performed based on CEA mRNA positivity, which is determined using a cutoff level [13]. CEA mRNA can be detected in patients with benign disease as well as healthy volunteers, so the cutoff levels are usually determined by maximum expression in non-malignant patients [18,19]. Setoyama T et al. found that the maximum value of CEA mRNA in patients without malignancy was 8.6, they therefore set the cutoff value as 9.0 [20]. Schuster R et al.[21] also used the maximum value of healthy volunteer background as the cut-off value for the CEA mRNA detection in colorectal cancer patients. In our study, we also used the maximum value of corrected CEA mRNA score in patients without malignancy as the cutoff value. By establishing a cutoff value of 100 for normalized CEA mRNA levels, we can distinguish cancer patients from non-cancer patients and, therefore, more confidently consider the expression of CEA mRNA as a marker of circulating tumor cells.

We found that 10 patients with T1 tumor, 6 patients had positive CEA-mRNA expression. But no record of recurrence was found in the 10 patients. It seems that there is no relationship between the CEA mRNA expression and recurrence in T1 tumor. It is hard to explain the high positive rate of CEA-mRNA in T1 patients, but we found that the CEA mRNA expression was low in the 6 T1 patients, ranging from 4320 copies to 44 600 copies. Ikeguchi M [22] reported that 12.5% of the stage I gastric carcinoma patients expressed CK20 mRNA and they considered that it was induced by a small CK20 expression in peripheral white blood cells.

Few reports have assessed the condition of CTCs in gastric carcinoma patients before treatment. Ikeguchi and Kaibara reported [23] that they could not find any cancer cells in peripheral blood from untreated gastric carcinoma patients. The authors speculated that cancer cells did not appear to easily migrate to the peripheral blood from primary tumors in patients with untreated gastric carcinoma. By contrast, Miyazono *et al. *[24] showed that the positive rate for CEA mRNA of gastric carcinoma patients was 8.8% before operation. The presence of CTCs before treatment and its relationship with clinical outcome thus remains controversial. In this study, we evaluated the clinical significance of CTCs in blood before operation by using real-time RT-PCR to detect expression of CEA mRNA. The positive rate of CEA mRNA before any treatment is 36.6%. Additional file 1 shows the positive rate of mRNA markers from literature for detection of tumor cells by real-time RT-PCR.

O'Sullivan *et al. *[25] suggested that preoperative detection of micrometastasis may reflect either transient shedding of cells, metastatic potential, or residual disease. In the present study, we found that CTCs were detected in blood before treatment in relation to recurrence. Several reports have demonstrated that preoperative detection of circulating cancer cells was a clear marker of poor patient survival, because many cases with circulating cancer cells preoperatively showed either extended lymph node metastasis or distant metastasis, thus the prognosis of such patients was poor [26-28]. In current study, we found that the expression of CEA mRNA was significantly related to disease recurrence. Furthermore, patients with positive CEA mRNA had shorter 3-year disease-free survival outcome. The incidence of recurrence was significantly higher in patients positive for CEA mRNA than in those negative.

The sensitivity of CEA mRNA expression to predict recurrence is only 56.8%. Nineteen of seventy-eight patients (24.4%) with negative CEA mRNA expression had tumor recurrence. Setoyama *et al. *[20] showed that 8 of 69 (11.6%) esophageal carcinoma patients with negative CEA mRNA expression had tumor relapse, and 6 patients had lymph node recurrence. One frequently used explanation of detection failure is that circulating cells are not homogeneously distributed and non-continuously shed into circulation [29,30]. Furthermore, the ideal marker (no illegitimate expression in blood, high expression in tumor cells) has not yet been found. Beyond CEA mRNA, other transcripts, including cytokeratin (CA) 18 [31], matrix metalloproteinase (MMP)-7 [32], CK 20 [33], Urokinase-type plasminogen activator receptor (uPAR), CK 19 and CK 7 [34], have been tried as potential markers of CTCs. However, the tumor cell shed should be a relatively rare event. Thus, whether peripheral blood is a suitable compartment for early detection of micrometastases is still controversial. Other compartments such as bone marrow or abdominal cavity are known to provide higher detection rates, probably due to a larger number of tumor cells present [35-37].

Another important issue is false positive expression of CEA mRNA. Twenty patients (44.4%) who had positive CEA mRNA expression did not record recurrence in the follow-up. One reason may be the relatively short follow-up period.

Alternatively, this may be quite reasonable because few carcinoma cells shed into the bloodstream succeed in establishing metastatic disease [25]. These circulating cancer cells might not attach to distant organs and might not grow. Recently, Méhes *et al. *[38] investigated the morphology of circulating cancer cells and found that the majority of circulating breast cancer cells was in a state of apoptosis. In the peripheral blood of cancer patients, the existence of the tumor cell-lymphocyte complex was observed [39]. These findings indicate that macrophages or lymphocytes could play an important role in the induction of circulating tumor cell apoptosis and the antitumor immune response of the host. These immunized macrophages may sensitize the cytotoxic T lymphocytes of the host, and the sensitized T lymphocytes may attack the residual micrometastatic cancer lesions of the patients [23]. To clarify this hypothesis, further investigations regarding the correlation between the existence of circulating cancer cells and the host immune response are necessary.

The current study was retrospective analysis, and patients who should receive adjuvant chemotherapy were not set ahead of time. Generally, patients who had positive lymph node were recommended to receive adjuvant chemotherapy. Till now, there are no standard criteria for adjuvant chemotherapy of gastric cancer in China. Our study showed that the CEA mRNA copy number in peripheral blood at initial diagnosis was significantly associated with disease recurrence in gastric adenocarcinoma patients. In the viewpoint of recurrence, we therefore suggest that patients who have positive CEA mRNA expression preoperatively receive adjuvant chemotherapy after radical resection.

## Conclusions

In this study, the sensitivity of CEA mRNA was higher than that of serum CEA. Moreover, according to multivariate regression analysis, CEA mRNA positivity was an independent factor for recurrence. The current study confirmed that such a method was promising for the early detection of CTCs in patients with gastric carcinoma before treatment; patients with positive CEA mRNA may have a higher risk of recurrence even after curative resection. However, a large randomized prospective study is warranted to define the role of CEA mRNA detection in blood.

## Abbreviations

RT-PCR: Reverse Transcriptase-Polymerase Chain Reaction; CTCs: Circulating Tumor Cells; CEA: Carcinoembryonic Antigen; CA19-9: Carbohydrate Antigen 19-9; TNM: Tumor-Node-Metastasis; AJCC: American Joint Committee on Cancer; GAPDH: Glyceraldehyde 3-Phosphate Dehydrogenase; MMP-7: Matrix Metalloproteinase-7; uPAR: urokinase-type Plasminogen Activator Receptor.

## Competing interests

We have no financial or personal relationships with other people or organizations that would bias our work. No benefits in any form have been received or will be received from a commercial party related directly or indirectly to the subject of our article.

## Authors' contributions

QMZ carried out the real-time RT-PCR, participated in the clinical data collecting of the gastric carcinoma patients and drafted the manuscript. LZH carried out the real-time RT-PCR. ZZW participated in the blood sample collecting. LYH performed the statistical analysis. WZQ and WFH participated in the design of the study. FA and WDY drafted the manuscript and participated in the statistical analysis. HP and XRH conceived of the study, and participated in its design and coordination and helped to draft the manuscript. All authors read and approved the final manuscript.

## Supplementary Material

Additional file 1 **Data from literature for detection of tumor cells by real-time RT-PCR of mRNA markers**. It shows the positive rate of mRNA markers from literature for detection of tumor cells by real-time RT-PCR. Click here for file
