# Supplementary material for: Detection of carcinoembryonic antigen messenger RNA in blood using quantitative real-time reverse transcriptase-polymerase chain reaction to predict recurrence of gastric adenocarcinoma
Source: J Transl Med. 2010 Oct 31;8:107. doi: 10.1186/1479-5876-8-107 (PMC2989934; doi:10.1186/1479-5876-8-107)
Supplement: Additional file 1 — Data from literature for detection of tumor cells by real-time RT-PCR of mRNA markers. It shows the positive rate of mRNA markers from literature for detection of tumor cells by real-time RT-PCR. [file 1479-5876-8-107-S1.DOC]

**Additional files**

**Additional file 1**

**Title: Data from literature for detection of tumor cells by real-time RT-PCR of mRNA markers**

**Description: It shows the positive rate of mRNA markers from literature for detection of tumor cells by real-time RT-PCR.**

| Studies | Samples | Diseases | Stage | Markers（mRNA） | Patients with positive blood samples (%) | Minimum threshold | **Methods to establish the threshold** |
| --- | --- | --- | --- | --- | --- | --- | --- |
| Koga T, et al. 2008 [1] | Peripheral blood | Gastric cancer | Curative patients | CK19 | 8/69 (11.6) | The maximum value of healthy volunteers. | The maximum value of healthy volunteers. |
|  |  |  |  | CK20 | 10/69 (15.5) |  |  |
| Tamura N, et al. 2007 [2] | Peritoneal washes | Gastric carcinoma | I-IV | CK20 | 26/164 (15.9) | 0.02 in CK20/GAPDH | Receive operating characteristic (ROC) curve |
|  |  |  |  | CEA | 43/164 (26.2) | 0.004 in CEA/GAPDH |  |
| Tetsuro Setoyama, et al. 2006 [3] | Peripheral blood | Esophageal squamous cell carcinomas | I-IV | CEA | 39/106(36.8) | 9.0 | The maximum value of CEA mRNA in patients without malignancy was 8.6 |
| Xu dong, et al. 2006 [4] | Peripheral blood | Colorectal cancer | Duke’s A-D | CEA | 34/169(35.8) | Not shown | Not shown |
|  |  |  |  | CK20 | 13/169(28.3) |  |  |
|  |  |  |  | CK19 | 62/169(41.9) |  |  |
| Yasuki Fujita, et al. 2006 [5] | Bone marrow | Gastric cancer | I-IV | CEA, CK19,CK20 | 1/65(1.5) | Not shown | The mRNA expression level of 1 cell in 1×10^7^ PBMCs as the threshold |
| Masahide Ikeguchi, et al, 2005 [6] | Peripheral blood | Gastric cancer after gastrectomy | I-IV | CEA | 27/59(46.0) | Not shown | Not shown |
| Yasuhiro Kodera, et al. 2005 [7] | Peritoneal washes | Gastric carcinoma | I-IV | CK20 | 145/230(63.0) | 0 | Arbitrarily employing the smallest detectable value as a cutoff value |
| Ronny Schuster, et al. 2004 [8] | Peripheral blood | Colorectal cancer | I-IV | Prot M, CEA, CK20 | 32/129(24.8) | Not shown | The maximum value of healthy volunteer background. |
| Yasuhiro Kodera, et al. 2002 [9] | Peritoneal washes | Gastric carcinoma | I-IV | CEA | 46/189 (24.3) | Not shown | Not shown |
| Hayao Nakanishi, et al. 2000 [10] | Peritoneal cavity | Gastric cancer | I-IV | CEA | 30/109(27.5) | 10. | The maximum value of CEA mRNA in negative control patients |

Reference

[1]. Koga T, Tokunaga E, Sumiyoshi Y, Oki E, Oda S, Takahashi I, Kakeji Y, Baba H, Maehara Y：**Detection of circulating gastric cancer cells in peripheral blood using real time quantitative RT-PCR.** *Hepatogastroenterology* 2008,55:1131-1135.

[2]. Tamura N, Iinuma H, Takada T: **prospective study of the quantitative carcinoembryonic antigen and cytokeratin 20 mRNA detection in peritoneal washes to predict peritoneal recurrence in gastric carcinoma patients.** *Oncol Rep* 2007, 17: 667-672.

[3]. Setoyama T, Natsugoe S, Okumura H, Matsumoto M, Uchikado Y, Ishigami S, Owaki T, Takao S, Aikou T: **Carcinoembryonic AntigenMessenger RNA Expression in Blood Predicts Recurrence in Esophageal Cancer.** *Clin Cancer Res* 2006,12:5972-5977.

[4] Xu D, Li XF, Zheng S, Jiang WZ: **Quantitative real-time RT-PCR detection for CEA, CK20 and CK19 mRNA in peripheral blood of colorectal cancer patients.** *J Zhejiang Univ Sci B* 2006, 7:445-451.

[5]. Fujita Y, Terashima M, Hoshino Y, Ohtani S, Kashimura S, Kanzaki N, Osuka F, Kogure M, Gotoh M: **Detection of cancer cells disseminated in bone marrow using real-time quantitative RT-PCR of CEA, CK19, and CK20 mRNA in patients with gastric cancer.** *Gastric Cancer* 2006,9:308-314. Epub 2006 Nov 24.

[6]. Ikeguchi M, Kaibara N: **Detection of Circulating Cancer Cells After a Gastrectomy for Gastric Cancer.** *Surg Today* 2005,35:436-441.

[7] Kodera Y, Nakanishi H, Ito S, Yamamura Y, Fujiwara M, Koike M, Hibi K, Ito K, Tatematsu M, Nakao A: **Prognostic significance of intraperitoneal cancer cells in gastric carcinoma: detection of cytokeratin 20 mRNA in peritoneal washes, in addition to detection of carcinoembryonic antigen.** *Gastric Cancer* 2005, 8:142-148.

[8]. Schuster R, Max N, Mann B, Heufelder K, Thilo F, Gröne J, Rokos F, Buhr HJ, Thiel E, Keilholz U: **Quantitative real-time RT-PCR for detection of disseminated tumor cells in peripheral blood of patients with colorectal cancer using different mRNA markers.** *Int J Cancer* 2004, 108: 219-227.

[9]. Kodera Y, Nakanishi H, Ito S, Yamamura Y, Kanemitsu Y, Shimizu Y, Hirai T, Yasui K, Kato T, Tatematsu M: **Quantitative detection of disseminated free cancer cells in peritoneal washes with real-time reverse transcriptase-polymerase chain reaction: a sensitive predictor of outcome for patients with gastric carcinoma.** *Ann Surg* 2002,235:499-506.

[10]. Nakanishi H, Kodera Y, Yamamura Y, Ito S, Kato T, Ezaki T, Tatematsu M: **Rapid quantitative detection of carcinoembryonic antigen-expressing free tumor cells in the peritoneal cavity of gastric-cancer patients with real-time RT-PCR on the lightcycler.** *Int J Cancer* 2000, 89:411-417.
